# Response to “The second shot to walled‐off necrosis: The sooner the better versus sooner or later”

**DOI:** 10.1002/deo2.187

**Published:** 2022-11-21

**Authors:** Rishi Pawa, Robert Dorrell, Swati Pawa

**Affiliations:** ^1^ Department of Medicine Section of Gastroenterology Wake Forest University School of Medicine Winston‐Salem North Carolina USA

We appreciate Sato et al's interest in our study. There has been conflicting evidence on the optimal timing of direct endoscopic necrosectomy (DEN).^1^ In a study by Yan et al., the number of necrosectomies required to achieve resolution was fewer in patients undergoing immediate DEN. In addition, in multivariable analysis, DEN at the time of initial lumen apposing metal stent placement was the only positive predictor for decreasing time to clinical success.^2^

One concern raised by Sato et al. was using the length of stay as a surrogate marker of treatment success. In our study, systemic inflammatory response syndrome was present in almost all patients at some point in the disease course (95% immediate DEN group and 100% delayed DEN group). However, patients with systemic inflammatory response syndrome within 1 week of drainage underwent dDEN. This group of patients not only had a shorter length of stay in the hospital after initial stent placement but also required fewer overall necrosectomies.

The authors requested additional markers of treatment success including time to clinical success and avoidance of DEN altogether. Clinical success was defined as a lack of symptoms and a decrease in collection size to less than 2 cm at which time the lumen apposing metal stent was promptly removed. The average time to clinical success was not statistically significant in the two groups (immediate DEN 40.0 days [SD 24.7] vs. delayed DEN 44.3 days [SD 24.0], *p* = 0.45). In addition, all patients in our study had necrosis greater than 10% and underwent at least one necrosectomy. A randomized controlled trial would further validate these results and eliminate biases innate to its retrospective design.

## CONFLICT OF INTEREST

Dr Rishi Pawa is a consultant for Boston Scientific.
